# The Predictive Validity of Individualised Load–Velocity Relationships for Predicting 1RM: A Systematic Review and Individual Participant Data Meta-analysis

**DOI:** 10.1007/s40279-023-01854-9

**Published:** 2023-07-26

**Authors:** Leon Greig, Rodrigo R. Aspe, Andy Hall, Paul Comfort, Kay Cooper, Paul A. Swinton

**Affiliations:** 1 School of Health Sciences, Robert Gordon University, Garthdee Road, Aberdeen, AB10 7QG UK; 2 Directorate of Psychology and Sport, University of Salford, Frederick Road, Salford, Greater Manchester UK; 3 Institute for Sport, Physical Activity and Leisure, Carnegie School of Sport, Leeds Beckett University, Leeds, UK; 4 Centre for Exercise and Sport Science Research, Edith Cowan University, Joondalup, Australia

## Abstract

**Background:**

Load–velocity relationships are commonly used to estimate one-repetition maximums (1RMs). Proponents suggest these estimates can be obtained at high frequencies and assist with manipulating loads according to session-by-session fluctuations. Given their increasing popularity and development of associated technologies, a range of load–velocity approaches have been investigated.

**Objective:**

This systematic review and individual participant data (IPD) meta-analysis sought to quantify the predictive validity of individualised load–velocity relationships for the purposes of 1RM prediction.

**Methods:**

In September 2022, a search of MEDLINE, SPORTDiscus, Web of Science and Scopus was conducted for published research, with Google Scholar, CORE and British Ethos also searched for unpublished research. Studies were eligible if they were written in English, and directly compared a measured and predicted 1RM using load–velocity relationships in the squat, bench press, deadlift, clean or snatch. IPD were obtained through requests to primary authors and through digitisation of in-text plots (e.g. Bland–Altman plots). Risk of bias was assessed using the Prediction model Risk Of Bias ASsessment Tool (PROBAST) and the review conducted in accordance with PRISMA-IPD guidelines and an a priori protocol. Absolute and scaled standard error of the estimates (SEE/SEE%) were calculated for two-stage aggregate analyses, with bootstrapping performed for sampling variances. Estimates were pooled using three-level hierarchical models with robust 95% confidence intervals (CIs). One-stage analyses were conducted with random intercepts to account for systematic differences across studies and prediction residuals calculated in the absolute scale (kg) and as a percentage of the measured 1RM. Moderator analyses were conducted by including a priori defined categorical variables as fixed effects.

**Results:**

One hundred and thirty-seven models from 26 studies were included with each identified as having low, unclear or high risk of bias. Twenty studies comprising 434 participants provided sufficient data for meta-analyses, with raw data obtained for 8 (32%) studies. Two-stage analyses identified moderate predictive validity [SEE% 9.8, 95% CI 7.4% to 12.2%, with moderator analyses demonstrating limited differences based on the number of loads (*β*_2Loads:>2Loads_ = 0.006, 95% CI − 1.6 to 1.6%) or the use of individual or group data to determine 1RM velocity thresholds (*β*_Group_:_Individualised_ =  − 0.4, 95% CI − 1.9 to 1.0%)]. One-stage analyses identified that predictions tended to be overestimations (4.5, 95% CI 1.5 to 7.4 kg), which expressed as a percentage of measured 1RM was equal to 3.7 (95% CI 0.5 to 6.9% 1RM). Moderator analyses were consistent with those conducted for two-stage analyses.

**Conclusions:**

Load–velocity relationships tend to overestimate 1RMs irrespective of the modelling approach selected. On the basis of the findings from this review, practitioners should incorporate direct assessment of 1RM wherever possible. However, load–velocity relationships may still prove useful for general monitoring purposes (e.g. assessing trends across a training cycle), by providing high-frequency estimates of 1RM when direct assessment may not be logistically feasible. Given limited differences in predictions across popular load–velocity approaches, it is recommended that practitioners opting to incorporate this practice select the modelling approach that best suits their practical requirements.

**Registration:**

https://osf.io/agpfm/.

**Supplementary Information:**

The online version contains supplementary material available at 10.1007/s40279-023-01854-9.

## Key Points

**Table:** 

Load–velocity-based 1RM predictions demonstrate a tendency to overestimate actual 1RM values. This bias may result in inappropriate load prescription when estimates are used to adjust the load lifted on a session-by-session basis.
There is currently no evidence of improved accuracy with more complex load–velocity-based models.
Given the errors associated with 1RM prediction, it is recommended that practitioners obtain direct assessments of 1RM. However, in instances where this is not possible, practitioners should select a modelling approach that best fits their own tolerance of error and relevant external factors (e.g. resources available, time, statistical understanding and ability to collect high-quality data with appropriate frequency), or consider alternative approaches such as monitoring the velocity measured against a standardised load.

## Introduction

Resistance training is considered the most effective means for enhancing a range of important physical qualities including maximum strength and power [1, 2]. Whilst effective programming of resistance training requires consideration of several variables (e.g. load, volume, effort, velocity, modality) and their interaction, current recommendations suggest that the most influential variable for inducing changes in maximum strength is the load lifted [3]. The load lifted is frequently prescribed on a relative scale and expressed as a percentage of the maximum load that can be lifted for a single, technically proficient, repetition (1RM). Prescribing relative loads in this manner facilitates both individualisation of the training stimulus and specification of various training zones thought to be appropriate for developing specific physical qualities [4]. In practice, it is common to directly assess an individual’s 1RM, requiring completion of an exercise across a series of incremental loads until a load that can be lifted only once with proper technique is identified [4]. However, despite both research and practical experience supporting both the reliability and efficacy of this method [5, 6], the process can be fatiguing, time-consuming and limited by the precision of a single measurement that may fluctuate due to changes in readiness, or trend substantively over the short-to-medium term due to changes in both fitness and fatigue [7].

Previous attempts to address limitations associated with direct assessment of 1RM include the use of indirect approaches, whereby 1RM values are estimated on the basis of various statistical models [8]. One popular approach includes measuring the maximum number of repetitions that can be performed with a submaximal load, which is then used to predict the individual’s 1RM using a range of previously validated regression equations that link the number of repetitions performed to the load lifted [9, 10]. While this method may be less time-consuming than a direct 1RM assessment, repeated administration of any repetition maximum test is likely to generate substantial levels of fatigue, thereby limiting the frequency with which the measurement process can be completed. Alternative methods that have grown in popularity include the use of load–velocity relationships [11] and the strong inverse linear relationship between the load lifted (expressed in both relative and absolute terms) and barbell velocity which has been repeatedly observed [11]. The increased popularity of these approaches is also partly due to the rapid proliferation in technologies capable of accurately measuring barbell velocity [12]. Unlike methods of direct assessment and repetition maximum testing, however, establishment of 1RM using load–velocity relationships may not require frequent participation in fatiguing protocols and can be readily integrated into pre-existing warm-up routines, meaning that the prediction of daily 1RM requires no additional time to complete [11, 13].

The seminal work of Gonzalez and Badillo [14] was the first to identify the potential in using load–velocity relationships for the purposes of daily resistance training load prescription. Since then, a range of approaches have been proposed for predicting 1RM from load–velocity relationships, with research indicating various degrees of predictive validity across a range of both upper and lower body exercises [15–25]. In general, these approaches involve development of regression models using velocity data gathered from an incremental loading protocol whereby individuals perform each repetition as quickly as possible but do not perform sets to a repetition maximum. An individual’s 1RM is then predicted by extrapolation of the regression equation to a velocity thought to represent the 1RM [11]. Whilst the majority of researchers have used the so-called minimum velocity threshold (the velocity associated with a previous 1RM), others have proposed using a velocity of 0 (LD0), or the velocity associated with the last repetition in a set performed to failure (*V*_last_). Representative approaches have also differed on a range of other factors, including the number of loads used to build the model, the function used to fit the data (e.g. polynomial versus linear) and the use of both group and individual data to obtain an MVT value [11]. While the strong relationship underpinning load–velocity approaches presents clear opportunities to prescribe loads in an effective and efficient manner, the broad range of approaches and current lack of robust evidence synthesis projects make it difficult to determine which approach should be recommended to balance accuracy and feasibility.

Previous studies investigating the predictive validity of load–velocity relationships have tended to adopt comparative designs whereby competing models are assessed through primary data collection. However, these approaches in isolation have yielded limited insight, as studies have often focused on only a selection of available approaches, whilst also employing a range of diverse—and often incompatible—criteria to describe predictive validity. For example, in a study by Jukic et al. [24], the authors described the predictive validity of different approaches in both absolute terms using the standard error of the estimate (SEE) and in relative terms using standardised mean differences. Whilst this approach provided more information, there were multiple instances whereby the two statistics provided different, and potentially conflicting, information. For one of the models examined, the SEE was reported to be 10.1 kg, and standardised mean difference zero. Additionally, in another model the SEE reduced from 10.1 kg to 5.7 kg, but the standardised mean difference increased from 0 to 0.11. These results highlight the challenge researchers face when selecting statistics which accurately convey information on model accuracy, whilst also providing results in a manner that are readily interpretable and practically relevant.

Previous attempts have also been made to synthesise existing load–velocity research through systematic [27] or narrative review [28]. However, no review to date has provided a robust quantitative synthesis of the predictive validity of common approaches. Previous reviews have attempted to gain insight through examination of model *R*^2^ values [27, 28]. Whilst the dimensionless nature of the *R*^2^ value is beneficial when comparing disparate models, the practical relevance is often difficult to identify. This is because the *R*^2^ value is principally a measure of model fit to observed data [29], meaning that models may display excellent *R*^2^ values whilst still yielding errors unlikely to be deemed acceptable in practice [29]. On the basis of the increasing research base and absence of robust quantitative approaches to synthesise information surrounding the use of load–velocity models for the purposes of 1RM prediction, the aims of this individual participant data (IPD) meta-analysis were threefold: (1) to quantitatively synthesise information from studies investigating the predictive validity of various load–velocity models; (2) investigate model-level characteristics that may influence predictive validity; and (3) report results in an informative manner that is more easily understood by practitioners who are most likely to use these procedures.

## Methods

This review was conducted in line with best practice guidelines for conducting systematic reviews, as outlined by JBI [30] and a pre-registered protocol (https://osf.io/agpfm/). Items were reported according to the PRISMA-IPD, which is a PRISMA variant specifically designed for IPD meta-analyses [31]. A completed version of the PRISMA-IPD checklist can be found in the supplementary materials (Online Resource 1). By selecting an IPD meta-analysis design, results from previous studies that were reported using diffuse statistics could be synthesised into common effect sizes both to facilitate further investigation and to enhance the interpretability of results.

### Search Strategy and Eligibility Criteria

Inclusion criteria for this review were developed according to the PIRD (Population–Index test–Reference test–Diagnosis of interest) mnemonic [32] detailed in Table 1. Given the non-medical nature of predictive models in this review, however, the ‘diagnosis of interest criteria’ was changed to ‘target variable of interest’. Studies were eligible for this review if they: (1) included participants of any sex, age and demographic with previous resistance training experience; (2) investigated Smith-machine or barbell variants of either the squat, bench press, deadlift, clean, clean and jerk, power clean, snatch or power snatch exercises; (3) conducted the index test (i.e. collection of load and velocity data used for prediction of 1RM) and reference test (measurement of criterion 1RM value) within 3 weeks of each other; and (4) directly compared measured 1RM with a predicted 1RM value generated from a load–velocity model. Studies were excluded from this review if they were not written in the English language or included participants with underlying health conditions, including those who had undergone substantive medical procedures in the past 6 months. Because of the focus of this review, studies including models that incorporated predictors other than velocity were excluded. Models were also excluded if data used to fit the model were inclusive of the 1RM load, meaning that 1RM estimates had to be obtained via extrapolation. In addition to study-level criteria, model-level criteria were also applied with models excluded where load–velocity data were collected following interventions designed to induce substantive fatigue, or where isolated contraction modes were investigated (e.g. eccentric only). These criteria were adopted to ensure the contexts and models included in this review reflected the procedures most often used when attempting to develop strength and power in athletes. The criteria and definitions used herein are largely in line with those stated in the pre-registered protocol (https://osf.io/agpfm/). However, minor changes such as inclusion of model-level criteria and amendments to the definitions presented in Table 1 were made to reflect the large range of contexts examined across the research base, and to provide clearer and more detailed definitions.

**Table 1:** PIRD inclusion criteria

Criteria	Definition
Population	Studies including individuals of any sex, age and demographic with previous resistance training experience and no underlying health conditions
Index test	Any regression model built using load–velocity data recorded at more than one load for the purposes of predicting 1RM through extrapolation in any of the following exercises: (1) squat, (2) bench press, (3) deadlift, (4) clean, (5) clean and jerk, (6) power clean, (7) snatch, (8) power snatch
Reference test	Any direct 1RM assessment whereby the outcome measure was the heaviest mass that could be lifted for a single repetition with appropriate technique
Target variable	Maximum strength as quantified by the measurement of an individual’s 1RM

A comprehensive search was conducted in three stages [30]. The first stage included a limited search of MEDLINE and SPORTDiscus databases using preliminary key search terms related to resistance training, prediction of 1RM and terms related to measurement of velocity (Online Resource 2). As no additional key words were identified, the initial strategy was used to conduct a full search including MEDLINE, SPORTDiscus, Web of Science and Scopus. Google Scholar, CORE, SportRxiv and British Ethos databases were also searched for unpublished literature, with searching completed on 10 September 2022. For all searches, key terms were combined with Boolean operators and search fields were restricted where possible to title and abstract only. No limitations were placed on date of publication. A full example of the search strategy can be found in Online Resource 2. All records were then imported into Proquest^®^ Refworks for de-duplication before being imported into the systematic review software Covidence (Melbourne, Australia). Relevant records were then identified using a two-stage process. First, the title and abstracts of all records were screened with irrelevant studies omitted. Full texts of studies identified as potentially relevant during title and abstract screening were then screened against the inclusion criteria for this review. A final search stage was then conducted which included both backwards (cited) and forwards (citing) citation tracking, combined with hand-checking of other relevant sources. For all stages of the screening process, records were screened independently by both L.G. and A.H., and disagreements resolved either through conversation or from input by a third and final reviewer (P.S.).

### Data Coding and Curation

IPD for each study were obtained through requests sent to primary authors and through digitisation of in-text plots illustrating differences between the measured and predicted 1RM. Data were digitised using the freely available WebPlotDigitizer software v4.5 (Ankit Rohatgi; https://apps.automeris.io/wpd/) and all digitising was performed in duplicate (L.G. and R.A.) to provide information on reliability of the process. Information relating to study demographics (e.g. sample size, relative strength, sex split), models investigated (e.g. number of loads used, extrapolation method, use of group- versus individualised-level data to make predictions) and exercises assessed was then extracted on a pre-piloted and standardised spreadsheet. Variables identified as potential moderators a priori were coded as a categorical variable, with levels assigned in alphabetical order. Number of loads was also treated as a categorical variable, with binary coding of either two loads or greater than two loads due to the extensive range used across studies (2–8) and, in some cases, the inability to determine the actual number of loads used.

### Risk of Bias

Risk-of-bias assessment for this review was completed using a modified version of the Prediction model Risk Of Bias ASsessment Tool (PROBAST), which is a validated risk-of-bias tool used when studies are designed to assess the predictive validity of statistical models [33]. This tool was selected as no exercise science equivalent currently exists, and factors such as blinding and randomisation which are frequently of importance under a causal framework were less relevant to the contexts examined in this review (i.e. within-subjects designs whereby the primary goal is prediction at the individual level). Instead, the factors most likely to be of importance include issues related to data curation, model selection and model building [34]. Before commencing risk-of-bias assessment, modifications were made to the existing PROBAST tool to account for the nature of models examined in this review. PROBAST was originally designed to evaluate multivariate models designed for purposes of either diagnosis or prognosis within medical settings. This differs from the models included in the current review which include only a single predictor (velocity). To ensure modifications were contextually relevant and suitably appraised the studies included in this review, a pilot trial was conducted by two researchers (P.S. and L.G.) with disagreements resolved through discussion. No further modifications were made, and risk-of-bias assessment was completed independently for all predictive models on a custom spreadsheet by two researchers (P.S. and L.G.). Disagreements were resolved through discussion in all instances.

### Statistical Analyses

The present study comprised both one-stage (simultaneous modelling of all individual data) and two-stage (aggregation of study-level effect size from individual data then traditional pooling) IPD meta-analysis approaches. Two-stage aggregate meta-analyses were conducted using the SEE (Eq. 1), and the SEE scaled by the reference test mean (SEE%). Both quantities were calculated for each load–velocity model by first estimating the SEE from model residuals (measured 1RM − predicted 1RM), and then subsequently scaling the resulting SEE estimate by the mean measured 1RM value for that study (i.e. the mean measured 1RM score for all participants). Uncertainty in SEE and SEE% estimates were calculated for each load–velocity model through bootstrapping of model residuals, and for each bootstrapped sample, calculation of the SEE and SEE%. The standard deviations of the bootstrap samples were then used as the within-study standard error of the calculated effect sizes (SEE and SEE%), as these are required for meta-analysis models [35]. Convergence of bootstrapped estimates were assessed through visual inspection of line plots to ensure the number of iterations selected was appropriate, with 1000 iterations initially selected. A final check was then completed by comparing the results obtained when bootstrapping was completed with an increased number of iterations (10,000). Calculated SEE and SEE% values and their corresponding within-study standard errors were then pooled using three-level hierarchical models, with random intercepts included to account for dependence in the outcomes synthesised [36]. In the main meta-analysis model conducted across all exercises, the SEE% was used to pool results. Exercise-specific meta-analyses models were then built using the raw SEE values, providing results in kg units to enhance interpretation. Cluster robust standard errors and associated 95% confidence intervals were produced for all meta-analytic models using the ClubSandwich package [37] for one-stage analyses and the Metafor package [38] for two-stage analyses. Parameters for all models were estimated using restricted maximum likelihood [39, 40].

1 $$\mathrm{SEE}= \sqrt{\frac{{\sum }{\left(\mathrm{measured}-\mathrm{ predicted }1\mathrm{RM}\right)}^{2}}{n-2}.}$$

One-stage analyses were performed though multilevel modelling of both scaled (expressed as a percentage of 1RM) and unscaled residuals (expressed in kg units). Data from all studies were incorporated simultaneously into three-level models with random effects included to account for the nested data structure inherent in this review. Prior to analyses, raw residuals were reflected where required by multiplying by − 1, such that a positive value indicated model overprediction. A new variable containing residuals scaled by the measured 1RM was then created to assess whether any differences in the magnitude of errors identified between exercises was influenced by the magnitude of the load lifted. As measured 1RM data were not available for all studies, these values were estimated from the difference between the measured and predicted 1RM as well as the mean of the measured and predicted 1RM, both of which were obtained from in-text figures (e.g. Bland–Altman). This was achieved by first halving the difference between the predicted and measured 1RM, and then subtracting the resulting value from the mean of the measured and predicted 1RM (obtained from in-text plots), as outlined in Eq. 2 below

2 $$\left(\frac{\mathrm{predicted }+\mathrm{ measured}}{2}\right)- 0.5 \left(\mathrm{predicted}-\mathrm{ measured}\right).$$

For both one-stage and two-stage analyses, intraclass correlation coefficients (ICCs) were calculated to quantify the covariance in multiple outcomes (models) reported from a single study. Conceptually, this statistic provides similar information to the *I*^2^ statistic when data are clustered [41]. Calculation of the ICC (Eq. 3) was made by dividing the between-study variance (level 3 variance) term by the sum of the variances across all levels as estimated from intercept-only models.

3 $$\mathrm{ICC}= \frac{{\sigma }_{\mathrm{between cluster}}^{2}}{{\sigma }_{\mathrm{Total}}^{2}}.$$

For both one-stage and two-stage analyses, meta-regressions of study-level variables identified a priori (https://osf.io/agpfm/) were performed using three-level mixed-effects models. In addition to the variables identified in the protocol, the influence of relative strength was also investigated as a study-level variable and was quantified by dividing the mean 1RM in the study by the mean body mass of the participants. Identification of relative strength as a potential moderator was based on previous observations that individuals with greater levels of relative strength tend to display velocity values that are comparatively lower than those observed in less experienced cohorts [42, 43]. This may result in larger error in 1RM estimations if predictions are based on models which use group-level data. Meta-regression with categorical predictors was conducted by selecting a reference category to compare with (i.e. *β*_reference_:_comparator_), with results presented such that *β*_reference_:_comparator_ > 0 indicates an increase in the error of predictions for the comparator relative to the reference. For moderator analyses involving continuous predictors (e.g. relative strength), meta-regressions were performed after mean centring. When investigating the influence of relative strength on predictive validity, the predictor was scaled to enhance interpretation of the regression coefficient. For both sets of analyses, moderator analyses were conducted only when there were at least four observations per level of the moderator variable [44]. Because each study could contribute more than one outcome to the analyses, an observation was defined as any model included in this review, except for relative strength, which was treated as a study-level variable. For both analyses, model appropriateness was assessed through visual inspection of the distribution of model residuals when plotted against the values fit by the model (Online Resource 3). All statistical analyses were conducted in the R environment with one-stage analyses performed using the lmerTest package [45] and two-stage analyses performed using the metafor package [46].

#### Reliability of digitisation and validity of digitised data

To assess potential errors during the digitisation of raw data, data were independently digitised by two reviewers (L.G. and R.A.) and reliability analyses conducted. All digitised data were recorded in a single spreadsheet and coded by study and model before pre-processing. Data were then sorted in descending order to account for differences in the order by which data were digitised, and rows with missing observations deleted. As the data were continuous, reliability was estimated through calculation of the typical error. This involved first calculating the difference between reviewer 1’s data and reviewer 2’s data for each model, and then subsequently calculating the standard deviation of the difference scores and dividing by $\sqrt{2}$ [47]. A similar process was also undertaken to assess the validity of the digitised data with comparisons made between a single set of digitised data and raw data obtained directly from study authors.

## Results

### Study Selection

**Fig. 1:**
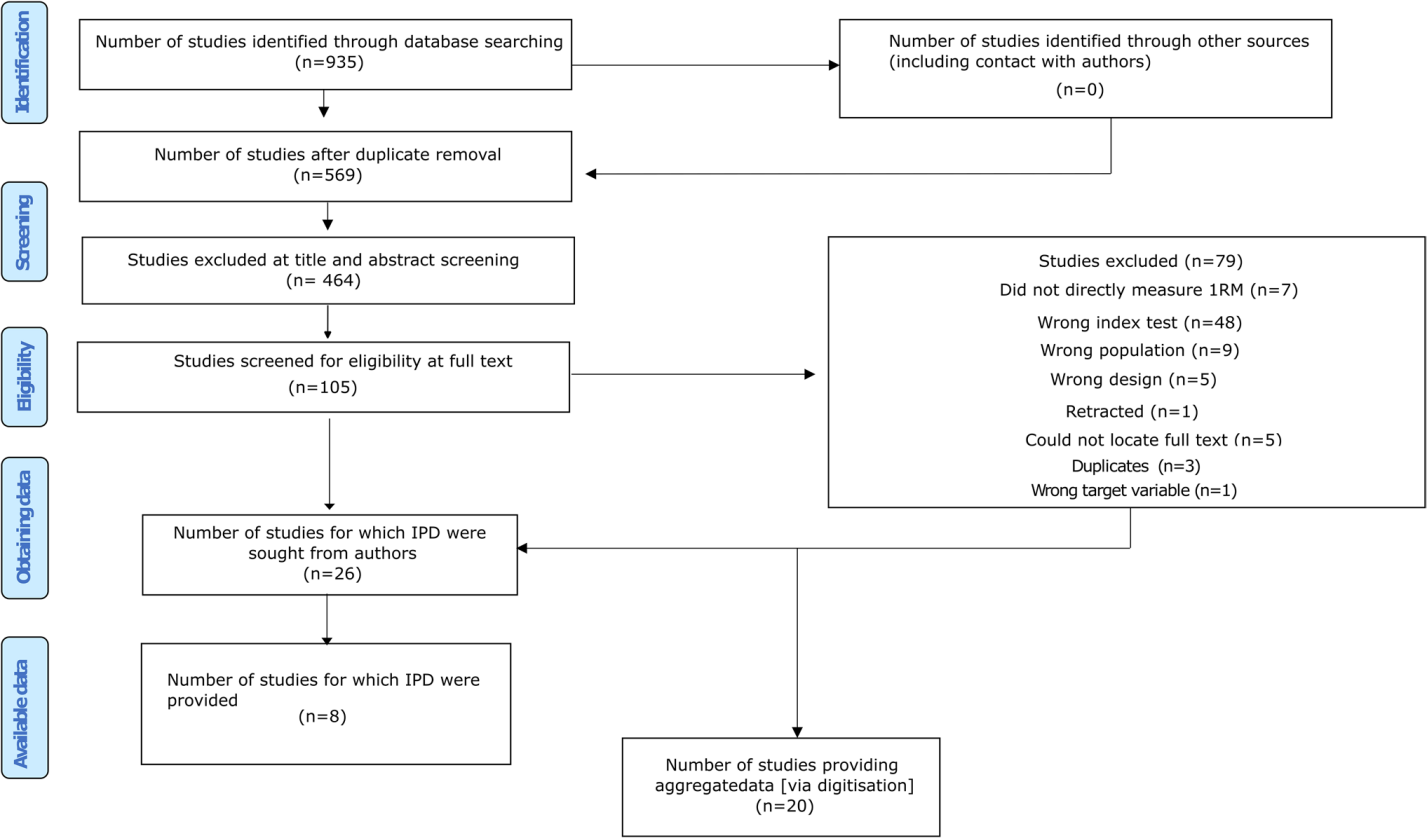
PRISMA-IPD flow chart demonstrating records at each stage of the systematic review process. *IPD* individual participant data

A total of 935 studies were identified from the search strategy, which reduced to 569 following removal of duplicates and 26 following screening of full texts against inclusion criteria. Broad reasons for study exclusion are listed in the PRISMA-IPD diagram (Fig. 1), and a more comprehensive list of reasons for each study can be found in Online Resource 4. Of the remaining 26 studies, a total of 20 (77%) contained figures enabling IPD extraction, with raw data provided by authors also obtained for 8 (31%) of the studies. Therefore, 20 studies (107 models) were eligible for quantitative synthesis, whilst 6 studies were eligible for narrative synthesis only. Across all studies included in either the quantitative or narrative synthesis, a total of 137 models were investigated across 641 participants.

### Risk of Bias

**Fig. 2:**
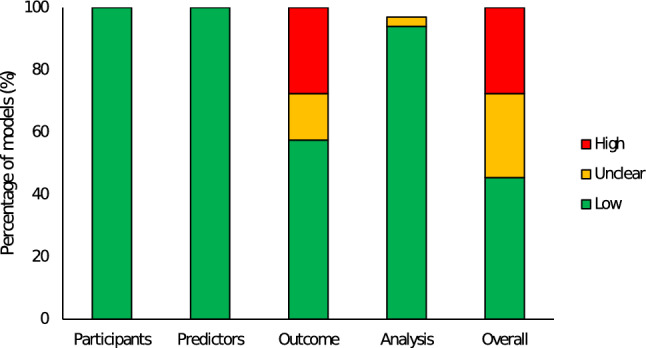
Stacked bar chart representing proportion of models (% of total) identified as low, unclear or high risk of bias across all domains, as assessed by the Prediction model Risk Of Bias ASsessment Tool (PROBAST)

A total of 61 (46%) of models were identified as having low risk of bias, with 36 (27%) and 37 (28%) models identified as having unclear or high risk of bias, respectively (Fig. 2). Downgrading from low to unclear or high risk of bias occurred when models used the velocity measured during the actual 1RM assessment as the MVT for predictions, or where this was unclear as per Sect. 3.3 of the PROBAST tool.

### Reliability and Validity of Digitised Data

Inter-rater reliability of digitised data quantified through mean typical error across all studies was 0.2 kg and ranged from 0.008 to 1.2 kg, indicating excellent reliability. Validity quantified by comparing digitised versus author provided data indicated good validity with the mean typical error across all studies being 0.11 kg, with range 0.01–1.1 kg. Bland–Altman analyses indicated a mean difference of 0.04 kg, when averaged across all models, suggesting that, whilst digitised data may have resulted in a slight bias, any overestimation is likely to be small and inconsequential given the overall modelling approach adopted. A more extensive overview of these results can be found in Online Resource 3, where both validity and reliability data are presented as the study median and range to account for the large number of models explored in some studies.

### Study Descriptors

The most common exercise assessed across studies was the bench press exercise, with 30 (22%) models investigated for the Smith machine bench press. The number of loads used to build load–velocity models in the included studies ranged from 2 to 8, with 2 most commonly used across all exercises (32%). One hundred and sixteen (87%) models predicted 1RM values using the MVT (% group, 63% individual), whilst 4 studies (15%) investigated other methods. Table 2 contains a breakdown of the number of participants per exercise alongside the mean 1RM strength and other model-related factors.

**Table 2:** Model characteristics of included studies

Exercise	Prevalence (number of models, number of studies)	Mean 1RM (± sd)	Load–velocity models		
			Number of loads (median: IQR)	Extrapolation/prediction method	Group versus individual data
Free-weight bench press	28 models across 9 studies	85.6 (46.3)	3 (3)	MVT (*n* = 28)	Group (*n* = 13) Individual (*n* = 9) NR (*n* = 6)
Smith-machine bench press	30 models across 5 studies	75.4 (8.9)	4 (2.5)	MVT (*n* = 24) NR (*n* = 2) Lasso (*n* = 1) Vlast (*n* = 3)	Group (*n* = 5) Individual (*n* = 21) NR (*n* = 4)
Back squat	42 models across 9 studies	145.5 (36)	4 (3)	MVT (*n* = 35), LD0 (*n* = 6), NR (*n* = 1)	Individual (*n* = 30) Group (*n* = 5) NR (*n* = 7)
Deadlift	29 models across 5 studies	165 (12.6)	4.5 (3)	MVT (*n* = 25), *V*_last_ (*n* = 4)	Individual (*n* = 14) Group (*n* = 9) NR (*n* = 6)
Power clean	4 models across 2 studies	NR	4.5 (1)	MVT (*n* = 4)	Group (*n* = 0) Individual (*n* = 0) NR (*n* = 4)

*MVT* minimal velocity threshold, *LD0* load at 0 velocity, *V*_*last*_ velocity of the last repetition during a set to failure, *NR* not reported, *Lasso* least absolute selection and shrinkage operator

### Two-Stage Aggregate Data Meta-analysis

#### Pooled Model (All Exercises)

**Fig. 3:**
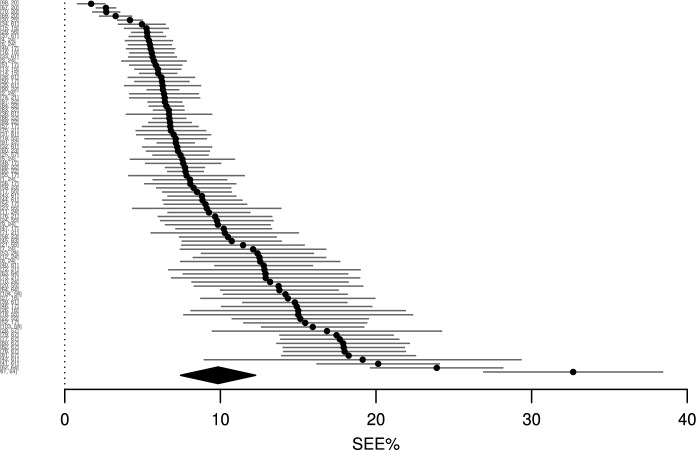
Caterpillar plot of all outcomes and corresponding 95% confidence intervals synthesised in the main two-stage meta-analyses model. Outcomes are plotted in ascending order according to the magnitude of the standard error of the estimate percentage (SEE%) (top-to-bottom). As each study could contribute more than one outcome (model), the y axis is labelled as “[Outcome, reference]” where outcome refers to the model number and reference is used to identify the study

The main two-stage meta-analysis model conducted across all exercises pooled 94 outcomes (bench press: 44; squat: 29; deadlift: 19; power clean: 2) from 18 studies indicated moderate predictive validity (SEE% 9.8, 95% CI 7.4% to 12.2%; Fig. 3), moderate within-study (${\sigma }_{2}$ = 2.3, 95% CI 1.8% to 3.1%) and between-study variation (${\sigma }_{3}$ = 4.5, 95% CI 3.1% to 6.8%) and substantial covariance between multiple measures reported from the same study (ICC 0.69). Moderator analyses indicated similar point estimates in predictive validity between bench press and deadlift with no significant differences identified (*β*_Bench:Deadlift_ =  − 2, 95% CI − 5.9 to 1.9%, *p* = 0.274). Greater relative error was observed in the point estimate for the squat compared with the bench press; however, this difference was also insignificant (*β*_Bench_:_Squat_ = 4.7, 95% CI − 2.8 to 12.2%,* p* = 0.196). Moderator analyses also indicated no significant differences between the use of group versus individualised MVT (*β*_Group_:_Individualised_ =  − 0.4, 95% CI − 1.9 to 1.0%, *p* = 0.515) or between using two versus multiple loads to predict 1RM (*β*_2Loads:>2Loads_ = − 0.006, 95% CI − 1.6% to 1.6%,* p* = 0.994).

#### Bench Press

The two-stage meta-analysis model for the bench press comprised a total of 44 outcomes pooled from 10 studies (median outcomes per study 3, range 1–14) and indicated moderate predictive validity (SEE 8.5, 95% CI 5.4 to 11.6 kg, *p* ≤ 0.001) and moderate within-study (${\sigma }_{2}$= 1.6, 95% CI 1 to 2.4 kg) and between-study variation (${\sigma }_{3}$= 4.0, 95% CI 2.4 to 7.3 kg), with substantial covariance observed between outcomes reported from the same study (ICC 0.76). Similar results were obtained when results were synthesised in standardised form (SEE% 9.9, 95% CI 6.8 to 12.9, *p* ≤ 0.001). Results from moderator analyses indicated no significant difference between the use of free weight or Smith machine in the prediction of 1RM (*β*_Free-weight:Smith_ =  − 3.6, 95% CI − 8.3 to 1.09 kg, *p* = 0.114), number of loads used (*β*_2loads:>2loads_ = 0.07, 95% CI − 1.1 to 1.2 kg, *p* = 0.888), or use of group versus individualised MVTs (*β*_Group:Individualised_ = – 0.08, 95% CI − 1.3 to 1.4 kg, *p* = 0.886). Mean centred meta-regression indicated a significant influence of relative strength (*β*_0_ = 0.75 kg, *β*_1_ = 0.98 kg, 95% CI 0.4 to 1.6 kg) with the slope parameter estimating that prediction errors would increase by 0.98 kg on average for each 0.1 unit increase in relative strength.

#### Deadlift

The two-stage meta-analysis model for the deadlift comprised a total of 19 outcomes pooled from 3 studies (median outcomes per study 6, range 1–12) and indicated poor predictive validity (SEE 13.3, 95% CI 9.5 to 17.1 kg, *p* = 0.004), large within-study variation (${\sigma }_{2}$= 4.01, 95% CI 2.0 to 7.63 kg) and negligible between-study variation (*σ*_3_ < 0.001, 95% CI < 0.001 to 3.16). Similarly, negligible covariance was observed for multiple outcomes reported from a single study (ICC < 0.001). Analyses conducted in terms of the SEE% indicated moderate predictive validity (SEE% 8, 95% CI 5.9 to 10.2, *p* = 0.004), with similar patterns observed for within-study (${\sigma }_{2}$ = 2.2, 95% CI 1% to 3.8%) and between-study (σ_3_ < 0.001, 95% CI < 0.001% to > 3.2%) variation. Insufficient data were available to perform moderator analyses investigating the influence of the number of loads, relative strength or the MVT used.

#### Squat

The two-stage meta-analysis model for the back squat comprised a total of 35 outcomes pooled from 7 studies (median outcomes per study 4, range 1–12) and indicated poor predictive validity (SEE 18.6, 95% CI 7.5 to 29.8 kg, *p* = 0.006). Moderate within-study variation (${\sigma }_{2}$ = 3.2, 95% CI 1.8 to 5.2 kg), substantial between-study variation (${\sigma }_{3}$ = 11.7, 95% CI 6.9 to 23.9 kg) and large covariance between multiple outcomes reported from the same study were also observed (ICC 0.9). Analyses conducted in terms of the SEE% also indicated poor predictive validity (SEE% 12.3, 95% CI 3.3% to 21.4%, *p* = 0.017. Insufficient data were available to perform moderator analyses investigating the influence of the number of loads, the MVT used or relative strength.

### One-Stage Meta-analysis

**Fig. 4:**
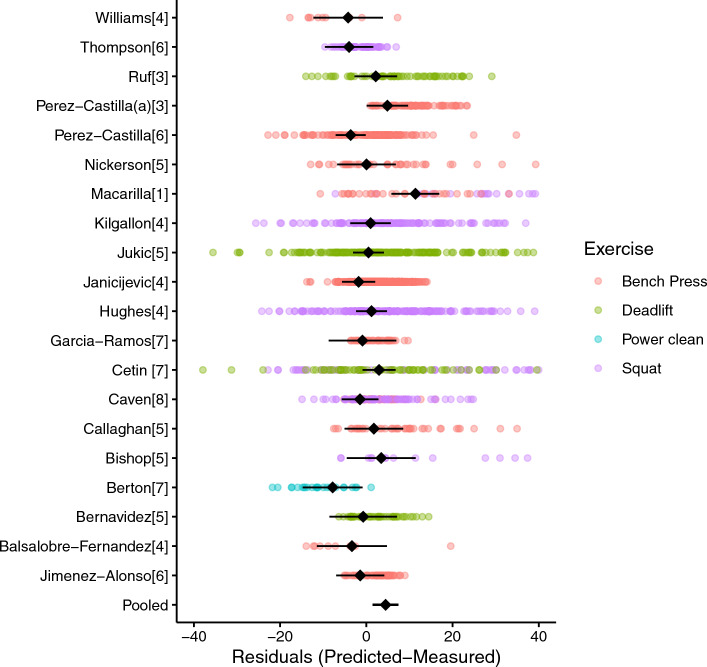
Plot of results from the main meta-analysis model conducted using unscaled residuals (kg) during one-stage analyses. Each individual data point represents the difference between the predicted and measured one-repetition maximum (1RM) for a single participant and model, where positive values represent overprediction, and negative values represent underprediction. Study “average” effects (best linear unbiased prediction) and associated confidence intervals (CIs) are included and represented as diamonds. The pooled estimate (i.e., model intercept) and cluster robust 95% CIs are included at the bottom and are represented as a diamond and whiskers

One-stage meta-analyses pooled across all exercises (Fig. 4) were initially conducted for both scaled (%1RM: 2289 observations from 19 studies) and unscaled (absolute load kg: 2355 observations from 20 studies) residuals. Analysis of unscaled residuals identified a small, albeit systematic overestimation of 1RM by 4.5 (95% CI 1.5 to 7.4 kg, *p* = 0.005), which, when scaled and expressed as a percentage of measured 1RM, was equal to 3.7 (95% CI 0.5% to 6.9% 1RM, *p* = 0.025). Moderate within-study (*σ*_2_ = 6.3, 95% CI 5.3 to 7.6 kg) and between-study (*σ*_3_ = 5.1, 95% CI 2.4 to 8.1 kg) variation was evident for unscaled residuals with moderate covariance between multiple outcomes reported from the same study also observed (ICC 0.36). Similar findings were obtained for variance parameters using scaled residuals (Online Resource 3). Moderator analyses with exercise included as a fixed effect indicated small prediction errors for the bench press (*β*_Bench_ = 2.7, 95% CI − 1.2 to 6.6 kg, *p* = 0.155). Relative errors were smallest for the deadlift (*β*_Bench_:_Deadlift_ =  − 1.5, 95% CI − 13.1 to 10.0 kg, *p* = 0.747), followed by the squat (*β*_Bench_:_Squat_ = 8.5, 95% CI − 3.6 to 20.6 kg, *p* = 0.133). Similar results were obtained for analyses reperformed using scaled residuals (Online Resource 3). Moderator analyses investigating the number of loads indicated small differences in predictive validity between models using only two loads in comparison with models using multiple loads when data were pooled across all exercises (*β*_Two_:_Multiple_ = − 1.5, 95% CI − 3.0 to − 0.1 kg, *p* = 0.038). A similar result was also observed when including the extrapolation method used as a fixed effect, indicating limited differences between the use of individualised or group level MVT data (*β*_Group_:_individual_ = 1.4, 95% CI − 2.0 to 4.9 kg, *p* = 0.301), with limited differences also observed for models using *V*_last_ (*β*_Group_:_Vlast_ = 0.8, 95% CI − 1.2 to 2.8 kg, *p* = 0.291). For both sets of moderator analyses, no substantial differences in results were obtained between models built using scaled versus unscaled residuals (Online Resource 3).

Of the six studies included for narrative synthesis only, each investigated a single exercise including the bench press [48, 49], back squat [50], deadlift [51] and power clean [39]. All six studies investigated free-weight variations and models built using mean concentric velocity (MCV); however, the studies by Lake et al. [50] and Haff et al. [51] also investigated the use of different velocity variables including mean propulsive or peak velocity, respectively. Despite large differences in models and exercises examined, there was a general trend for models to overestimate an individual’s 1RM. For example, Banyard et al. [49] reported systematic errors ranging from 10 to 17 kg during the back squat, whilst Medrano et al. [47] reported errors comparatively smaller in magnitude ranging from 7 to 9 kg in the bench press. Lake et al. [50] also identified a general trend for models to overestimate 1RM in the deadlift, with mean differences ranging from 16 to 28 kg. In contrast to the other studies included in this review, however, Lake et al. [38] used the velocity measured during the last successful repetition of a set completed to failure to represent the MVT in their predictions. The authors also observed greater predicative validity when models were built using mean propulsive velocity, as opposed to mean concentric velocity. To date, only two studies have investigated the predictive validity of load–velocity models for use with commonly used weightlifting exercises and their derivatives [51, 52]. In the study by Haff et al. [51], the authors compared several models differing in either the number of loads (3 or 4) or the velocity metric used (MCV or peak velocity) for predicting the power clean 1RM. Despite identifying that the three-load peak velocity model resulted in the best predictions, some evidence of underestimation was observed across all models with no significant differences identified in model performance when stratified according to participants’ maximum strength. These results are in line with the findings by Berton et al. [52] who also identified a general trend to underestimate by approximately 4–6.5 kg when models were built using peak velocity data gathered across several loads.

## Discussion

The present review is the first to employ a robust quantitative synthesis of information relating to the validity of load-velocity models for predicting 1RM. The results highlight a general trend for all load–velocity-based models to systematically overestimate an individual’s measured 1RM with no significant differences in predictive validity identified across the modelling approaches included. Results from one-stage analyses estimated a 4.3 kg mean error in prediction across all models, which expressed as percentage of the measured 1RM was equal to 3.6%. Limited differences in predictive validity were observed when investigating the influence of group-level versus individualised-level MVTs, or when comparing the use of two versus multiple loads to build predictive models. Differences in model performance, however, were observed on the basis of the exercise investigated, with the back squat demonstrating the largest errors and the bench press demonstrating the smallest errors.

The primary findings generated from the current review are in general agreement with those reported from many individual studies, which is an overestimation of an individual’s 1RM, irrespective of the modelling approach selected [15, 16, 23, 24, 49, 53–55]. Despite systematic overestimation being a common finding, the magnitude of these errors appears to be influenced by the exercise selected. In the current review, moderate yet insignificant differences were observed between the bench press and squat exercises (*β*_Bench_:_Squat_ = 8.5, 95% CI − 3.6 to 20.6 kg), with smaller differences also observed between the bench press and deadlift (*β*_Bench_:_Deadlift_ =  − 1.5, 95% CI − 13.1 to 10.0 kg).These trends were evident during both one-stage and two-stage analyses and are congruent with the general range of errors reported across studies. For example, studies investigating the back-squat exercise have frequently reported errors as large as 10–20 kg [49], whilst studies investigating the deadlift and bench press exercises have tended to report errors comparatively lower in magnitude and within the 2–10 kg range [23]. It is reasonable to expect that absolute errors in 1RM prediction will be influenced by the magnitude of the 1RM loads lifted, and this may offer a partial explanation for the disparity observed in model performance. However, analyses conducted on scaled residuals (such that errors were expressed as a percentage of the measured 1RM), were consistent with results obtained during analysis of unscaled residuals for both the squat (*β*_Bench_:_Squat_ = 4.5, 95% CI − 8.0 to 17.1%) and the deadlift (*β*_Bench_:_deadlift_ = − 3.2, 95% CI − 14.9 to 8.5%), suggesting that the differences observed between exercises may not be explained by the magnitude of the loads lifted. Across all exercises, prediction errors expressed as percentage of 1RM were in a range likely deemed acceptable by most practitioners (~ 4–6% of measured 1RM). However, regardless of whether residuals were expressed in absolute or relative terms, analyses demonstrated that overestimation was most likely to occur. This overestimation suggests that current models are limited by the profile of the regression such that non-linearities and concave features at the upper range are underappreciated, and/or identification of the 1RM velocity is overestimated. Indeed, despite studies providing consistent evidence that linear relationships describe the relationship between loads lifted and barbell velocity very well, some authors have shown that improvements in model fit can be obtained through non-linear modelling [56]. For example, Pestana-Melero et al. [56] compared linear and polynomial regression models when modelling the load–velocity relationship in the Smith machine bench press exercise. The authors described model fit through assessment of median *R*^2^ values and their associated ranges, observing an increased median *R*^2^ value (0.995) and tighter range (0.985–1.00) for polynomial models in comparison with their linear counterparts (*R*^2^ = 0.990, range 0.964–0.998). However, these differences are small and not necessarily indicative of practically relevant increases in model performance [24]. In fact, multiple comparative studies have now shown no improvements in predictive validity when comparing polynomial models with the linear counterparts across a range of exercises, including the bench press [22], deadlift [24] and squat [21]. Given the consistent evidence of overestimation associated with load–velocity relationships, and evidence that polynomial formulations may provide limited improvements in predicative validity, future research may seek to explore additional modelling functions that may better capture underlying relationships, and/or identify alternative methods for selecting the MVT to predict 1RMs. Additional sources of error that are likely to influence the predictive validity of the derived 1RM include measurement error inherent to the process, which may comprise both biological and instrumental components [47] However, unlike the systematic sources identified in this review, these are likely to be random in nature, and can be minimised through selection of devices with established validity and reliability, as well as standardisation of data collection protocols [47].

Common to all models included in this review was the use of extrapolation to generate 1RM predictions. This process requires selection of a velocity outside the range of the measured data to a point thought to represent an individual’s 1RM. Researchers have most often selected this value by using the velocity recorded during a previous 1RM lift under controlled conditions [17, 23, 49], and have proposed using both group-level (i.e. mean 1RM velocity across all participants) or individual-level data [11]. However, researchers have also proposed generating predictions through extrapolation to the load which would produce a velocity of 0 [17, 57] and, less commonly, through extrapolation to the velocity recorded during the final repetition of a set performed to failure (*V*_last_) [28, 50, 58]. In the current review, limited differences in model performance were observed when comparing the use of group versus individualised MVTs during both one-stage (*β*_Group_:_individual_ = 1.4, 95% CI − 2.0 to 4.9 kg) and two-stage (*β*_Group_:_Individualised_ =  − 0.4, 95% CI − 1.9% to 1.0%) analyses. Whilst both the magnitude of errors observed and a lack of evidence for differences in predictive validity based on the MVT used are in general agreement with previous observations [24, 59], conflicting evidence does exist, with two studies suggesting the use of individualised MVTs may be inappropriate [17, 49]. Both Banyard et al. [49] and Hughes et al. [17] compared a range of load–velocity models for predicting an individual’s 1RM in the back squat using individualised MVT, with both authors reporting substantial model overestimation (≥ 10 kg). In addition, Banyard et al. [49] also documented unacceptable changes in the velocity associated with an individual’s 1RM between adjacent trials (coefficient of variation 22.5%), concluding that models based on this metric were generally unreliable and unlikely to yield valid estimates. Because neither study directly compared individualised MVTs to group MVTs, it is unclear whether group MVTs may have displayed errors comparatively large in magnitude when examined under similar testing conditions. In contrast, Jukic et al. [24] directly compared the predictive validity of 1RM estimates generated using either group- or individual-based MVTs. The authors observed limited differences in model performance and concluded that group MVTs may still be preferable for enhancing the ease with which these procedures could be implemented [24]. Given the general agreement in findings between this review and previous individual studies, it is plausible that the discrepancies reported by both Banyard et al. [49] and Hughes et al. [17] are attributable to factors other than the MVT selected, including the participants used, procedures implemented and exercise investigated as well as the technology and its calibration. To better establish the validity across different MVT thresholds and approaches, future research implementing well-designed comparative studies which standardise factors thought to influence model performance is required.

Initial research investigating the use of load–velocity relationships for 1RM prediction frequently investigated models with more than two loads [17, 23, 24, 47, 50, 51, 53, 59–63]. However, given the increased popularity of these procedures and the desire to implement them across different practical environments, including those encompassing the simultaneous training of many athletes, researchers suggested that valid 1RM estimates could be obtained more efficiently using two evenly spread loads—one from each end of the relationship (e.g. 40% and 90% 1RM). Previous recommendations have generally advised that a minimum velocity difference of 0.5 m/s should be observed between the two loads selected to ensure sufficient coverage of the underlying relationship is achieved [57]; however, the actual difference in velocity required is likely both exercise and individual specific. Preliminary research by Garcia-Ramos et al. [64] was the first to provide support for this premise, identifying limited differences in the predictive validity when models were built using two loads in comparison to four loads during the free-weight bench pull. These findings have since been replicated with other exercises [24, 65] and are supported by the results of the current review. While initial consideration may have expected that increasing the number of loads (and therefore observations) would generate more accurate predictions [66], what is likely to be of most importance is the spread of the loads included [i.e. including loads at both the low (30%) and high (90%) end of the range]. This is because most models investigated rely on the process of extrapolation, whereby predictions are obtained through extension of the modelled relationship beyond the range of observed data. Implicit in this process is the assumption that modelled data are representative of the underlying relationship, meaning that estimates derived from models which capture the majority of the relationship are most likely to result in the least error [66]. Practically, this means including loads as close to the upper range of the relationship as possible is desirable for maximising the validity of predictions. Studies comparing models with only two loads have most often selected a similar spread in loads recorded for both the ‘two-point’ models (e.g. 40% and 90%) and models incorporating multiple loads. Therefore, the limited differences observed between the two modelling approaches both in research and in this review are likely in part due to the studies investigating two-point models capturing a comparable range of the relationship to those investigating models incorporating multiple loads (e.g. 40%, 60%, 80%, 90%).

One area that has received less attention in the literature is whether differences in predictive validity exist based on the mode of resistance selected. Only two studies included in this review examined exercises performed using a Smith machine [67]. This is likely reflective of practice where the majority of compound movements are performed using free-weight equipment [68]. The results of this review support previous studies, identifying a lack of evidence for a difference in predictive validity between free-weight and Smith-machine variants of the bench press exercise. In the current review results were consistent across both two-stage (*β*_Free-weight:Smith_ =  − 3.6, 95% CI − 8.3 to 1.1 kg) and one-stage analyses performed on both scaled (*β*_Free-weight:Smith_ =  − 1.13, 95% CI − 10.5% to 8.3% 1RM) and unscaled data (*β*_Free-weight:Smith_ = 0.5, 95% CI − 8.6 to 9.6 kg). Despite consistency in findings across research, initial considerations may have expected greater errors with free-weight movements due to larger degrees of medio-lateral and/or anteroposterior displacement [11, 68] which linear position transducers are generally unable to measure. However, the results of this review do not support this hypothesis. One potential explanation for this is that all the included studies comparing Smith-machine and free-weight variants of an exercise have investigated the bench-press exercise where absolute errors are already small, and thus any differences between modalities are likely to be less pronounced than in exercises with larger non-vertical components of displacement. Owing to a lack of studies using Smith machine variants of other exercises included in this review, it was not possible to identify if discrepancies between modalities may exist for exercises such as the squat and deadlift. Whilst the paucity of literature is likely reflective of the tendency to use free-weight variations of these exercises in practice, coaches may still wish to remain cognisant that predictions generated using velocity data collected during free-weight exercises with substantial medio-lateral and/or anteroposterior components may produce larger disparities between the free-weight and Smith-machine variants than those reported both throughout the literature and in this review. This is particularly likely to be the case where velocity data are collected using devices with poorer validity and reliability (e.g. accelerometer-based instruments) or devices that are incapable of capturing non-vertical components of displacement (e.g. LPTs).

Adopting an IPD approach enabled quantitative synthesis where previous reviews were limited to narrative syntheses only. There are, however, several limitations of the current review that should be considered and can broadly be categorised into limitations arising from the methods adopted and the included studies. In the current review sampling variances for each outcome were estimated through bootstrapping of model residuals. While bootstrapping is an approach commonly used to estimate distributional properties, the procedure is also dependent on the samples used [69]. Owing to the small sample sizes presented across the majority of included studies, it is possible that modelled estimates obtained for within-study variation may lack accuracy, and—as a result—may have influenced associated confidence intervals and the weighting of individual studies during two-stage analyses [70]. To address this, the stability of estimates was investigated increasing sample sizes from *n* = 1000 to *n* = 10,000 with no substantive differences identified. A second limitation is that it was not possible to obtain raw data for all studies identified in the search for this review. In such instances, data were obtained through manual digitisation of in-text figures, and subsequently synthesised. Substantial inaccuracies in the digitisation process could lead to poor estimates influencing findings from analyses. Reliability and validity assessments, however, indicated that the processes adopted were likely to produce stable and sufficiently accurate data. A third limitation of the current review includes an inability to investigate moderators that are likely to be of practical interest, such as the difference between polynomial and linear models. The inability to perform these analyses stemmed primarily from the large degree of heterogeneity between studies, meaning that in many cases there were insufficient data at each level of the moderator to enable robust analyses. In addition, the nature of data obtained for this review meant that identification and coding of each individual participant was often not possible, and therefore assessment of individual-level characteristics such as relative strength levels which may better explain some of the disparities observed was precluded. One salient limitation of the included evidence, and—by extension—this review, is the use of previously assessed 1RM values as a comparator against which 1RM predictions are assessed. This is problematic as it results in an inability to identify whether the observed error in 1RM predictions stems from the modelling approach selected, or a real change in an individual’s 1RM since the last measurement occasion. Therefore, to ensure that inferences regarding predictive validity are well founded, future research should ensure that predictions are assessed against a 1RM measured on the same day/session as the velocity data used to build the model are collected. A final limitation of this review pertains to the presentation of results and their interpretation from a practical standpoint. An aim of the current review was to quantitatively synthesise the existing literature whilst presenting results in units directly interpretable by those most likely to use these approaches in practice. During the two-stage modelling approach, results were presented in standardised units as the SEE% to account for a broad range of differences across the outcomes synthesised. Whilst the SEE% is more easily interpretable than more commonly used effect size measures (e.g. Cohen’s *d*), the statistic is symmetrical, meaning that it provides limited insight into whether errors are chiefly systematic or random in nature. This information is likely to be of importance to practitioners, as overestimation is likely to be deemed less favourable than underprediction, even when the magnitude of these errors is equivalent. These results were therefore supplemented using a one-stage IPD meta-analysis, whereby outcomes were incorporated simultaneously using multilevel modelling. Adopting this approach allowed for the data to be synthesised in their raw units and for results to be presented in a practically relevant format using both kg and %1RM units.

## Conclusion

It was identified earlier in this review that a principal limitation of existing literature was the range of different criteria adopted to describe model performance. Not only has this meant that the practical relevance of primary research has often been difficult to identify, but it has also hampered the number of evidence synthesis projects attempting to quantitatively summarise the literature. Given the broad range of statistics previously used to evaluate model performance, future research may seek to adopt an approach similar to this review, whereby results are presented both in absolute terms (e.g. kg) and in standardised (e.g. SEE%) units. Not only would this increase the practical relevance of research, but it may also facilitate future evidence synthesis projects. Researchers may also wish to explore additional factors influencing model performance such as the spread of loads used. Whilst some evidence may already provide limited insight into the spread of loads used, firm conclusions cannot be made as changes in the spread of loads often occur concurrently with changes in other characteristics such as the number of loads used. Therefore, future researchers should ensure they adopt well-designed comparative studies whereby the independent variable is manipulated whilst other key model characteristics (e.g. exercise assessed or number of loads used) are held constant across conditions.

On the basis of limited evidence favouring any one load–velocity approach to predict 1RM, practitioners seeking to obtain higher-frequency 1RM estimates should select the approach that best suits them, their error tolerance and the individual needs of their athletes. This means that suitable 1RM predictions can likely be derived using a minimum of two evenly spread loads (e.g. 40% and 85%1RM) with a group average MVT used as the point of extrapolation. However, on the basis of the evidence available thus far, practitioners adopting this approach may also wish to calculate MVT values based on group averages from the population of interest, instead of adopting the values used in previous studies, where the population may not be representative. Practitioners adopting these methods for the purposes of daily 1RM estimation and subsequent load prescription should also remain cognisant of the likely systematic overestimation evident across all methods. While the magnitudes of these errors appear to be—on average—acceptable, it is likely that the systematic overestimation of loads is less desirable than systematic underestimation, and that such errors may lead to undesirable training effects if training prescriptions are consistently and/or frequently made on the basis of these unadjusted estimates. Practitioners may also wish to explore alternative VBT methods for monitoring fluctuations in performance such as the assessment of movement velocity against a standardised load which may be more feasible.

## Supplementary Information

Below is the link to the electronic supplementary material.

Supplementary file1 (XLSX 25 KB)

Supplementary file2 (DOCX 38 KB)

Supplementary file3 (DOCX 14 KB)

Supplementary file4 (DOCX 81 KB)

## Declarations

### Availability of data and material

Aggregate data are available on the Open Science Framework, and can be accessed using the following link https://osf.io/6dxp5/.

### Code availability

All code produced as part of this review can be found at the following link https://osf.io/6dxp5.
