# Supplementary material for: The Predictive Validity of Individualised Load–Velocity Relationships for Predicting 1RM: A Systematic Review and Individual Participant Data Meta-analysis
Source: Sports Med. 2023 Jul 26;53(9):1693–708. doi: 10.1007/s40279-023-01854-9 (PMC10432349; doi:10.1007/s40279-023-01854-9)
Supplement: Supplementary file 3 — Supplementary file3 (DOCX 14 KB) [file 40279_2023_1854_MOESM3_ESM.docx]

|  | **Search string** |
| --- | --- |
| 1. Velocity | **AB/TI:** velocity |
| 1. Prediction | **AB/TI:** predict* OR estimat* |
| 1. 1RM | **AB/TI:** 1RM OR 1-RM OR “repetition maximum” |
| 1. Combined string | S1 AND S2 AND S3 |
