# Supplementary material for: The Predictive Validity of Individualised Load–Velocity Relationships for Predicting 1RM: A Systematic Review and Individual Participant Data Meta-analysis
Source: Sports Med. 2023 Jul 26;53(9):1693–708. doi: 10.1007/s40279-023-01854-9 (PMC10432349; doi:10.1007/s40279-023-01854-9)
Supplement: Supplementary file 4 — Supplementary file4 (DOCX 81 KB) [file 40279_2023_1854_MOESM4_ESM.docx]

**Table 1:** Reliability of the digitisation process as assessed through the typical error (kg).

| **Study** | **Median (kg)** | **Range (kg)** |
| --- | --- | --- |
| Jukic et al. | 0.05 | 0.02 - 0.12 |
| Jiménez-Alonso et al. | 0.027 | 0.02 - 0.04 |
| Caven et al. | 0.02 | 0.01 – 0.03 |
| Williams et al. | 0.048 | 0.04 |
| Nickerson et al. | 0.12 | 0.12 |
| Hughes et al. (a) | 0.082 | 0.03 - 0.1 |
| Bishop et al. | 0.11 | 0.11 |
| Balsalobre-Fernandez et al. | 0.23 | 0.23 |
| Perez-Castilla et al. | 0.034 | 0.02 - 0.4 |
| Hughes et al. (b) | 0.09 | 0.05 - 0.1 |
| Ruf, Chery and Taylor | 0.03 | 0.03 - 0.04 |
| Macarilla | 0.1 | 0.06 – 0.17 |
| Callaghan et al. | 0.06 | 0.058 - 0.062 |
| Perez-castilla (b) (471) | 0.22 | 0.05 – 0.37 |
| Kilgallon et al. | 0.24 | 0.18 – 0.32 |
| Janicijevic et al. | 0.18 | 0.09 – 1.2 |
| Cetin et al. | 0.5 | 0.13 – 0.92 |
| Berton et al. | 0.08 | 0.07 – 0.09 |

**Table 2:** Validity of the digitisation process as assessed through the typical error (kg)

| **Study** | **Median (kg)** | **Range (kg)** |
| --- | --- | --- |
| Jukic et al. | 0.04 | 0.01- 0.05 |
| Caven et al. | 0.06 | 0.03-1.1 |
| Bishop et al. | 0.05 | 0.05 |
| Perez-Castilla et al. | 0.03 | 0.03 |
| Balsalobre-Fernandez et al. | 0.03 | 0.12 |
| Thompson et al. | 0.02 | 0.02 – 0.03 |
| Kilgallon et al. | 0.14 | 0.1 – 0.22 |
| Janicijevic et al. | 0.14 | 0.09 – 0.2 |

**Table 3:** Validity of digitisation process as assessed through mean difference of raw and digitised data

| **Study** | **Median (kg)** | **Range (kg)** |
| --- | --- | --- |
| Jukic et al. | 0.03 | -0.22 to 0.25 |
| Caven et al. | 0.03 | -0.35 to 0.19 |
| Bishop et al. | 0.12 | 0.12 |
| Perez-Castilla et al. | -0.02 | -0.02 |
| Balsalobre-Fernandez et al. | 0.12 | 0.24 to 0.26 |
| Thompson et al. | -0.01 | -0.0 to -0.014 |
| Kilgallon et al. | -0.004 | -0.2 – 0.37 |
| Janicijevic et al. | 0.02 | -0.04 – 0.04 |

**Model Diagnostics**

**Figure 1**: Plot of model residuals regressed on model fitted values for one stage intercept only model incorporating raw residuals (kg)


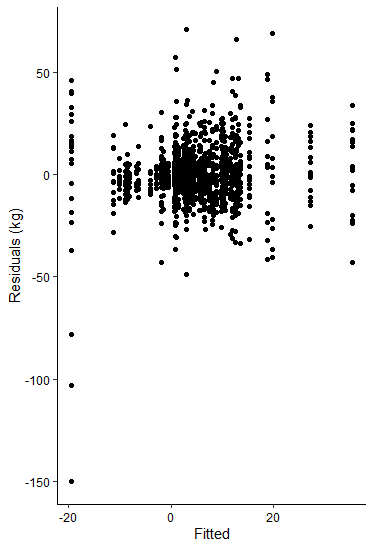


**Figure 2:** Plot of model residuals regressed on model fitted values for one stage intercept only model incorporating scaled residuals (%1RM)


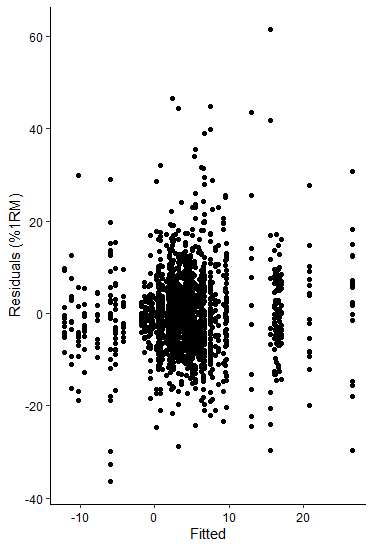


**One stage analyses supplemental results**

**Table 1:** Results from intercept only models incorporating both scaled and unscaled residuals

| **Residuals (kg)** | | | | | **Residuals scaled (%1RM)** | | | |
| --- | --- | --- | --- | --- | --- | --- | --- | --- |
| Predictors | Estimates | | 95% CI | *p* | | Estimates | 95%CI | *p* |
| Intercept | 4.5 | | 1.5 to 7.4 | 0.005 | | 3.7 | 0.5 to 6.9 | 0.025 |
|  |  | |  |  | |  |  |  |
|  |  | |  |  | |  |  |  |
| **Random effects** | | | | | | | | |
| σ | | 10.8 | | | 8.2 | | | |
| τ_within_ | | 6.3 | | | 4.4 | | | |
| τ_between_ | | 5.1 | | | 5.9 | | | |
| Observations | | 2355 (107models; 20 studies) | | | 2289 (101 models; 19 studies) | | | |

**Table 2:** Results of moderator analyses investigating the influence of exercise on model accuracy using both scaled and unscaled residuals.

| **Residuals (kg)** | | | | | **Residuals scaled (%1RM)** | | | |
| --- | --- | --- | --- | --- | --- | --- | --- | --- |
| Predictors | Estimates | | 95% CI | *p* | | Estimates | 95%CI | *p* |
| Bench (Intercept) | 2.7 | | -1.2 to 6.6 | 0.155 | | 3.5 | -1.0 to 8.1 | 0.114 |
| Deadlift | -1.5 | | -13.1 to 10.0 | 0.747 | | -3.2 | -14.9 to 8.5 | 0.453 |
| Squat | 8.5 | | -3.6 to 20.6 | 0.133 | | 4.5 | -8.0 to 17.1 | 0.368 |
| **Random effects** | | | | | | | | |
| σ | | 10.8 | | | 8.2 | | | |
| τ_within_ | | 5.5 | | | 4.0 | | | |
| τ_between_ | | 5.8 | | | 5.5 | | | |
| Observations | | 2331 (105 models; 19 studies) | | | 2265 (99 models; 18 studies) | | | |

**Table 3:** Results of moderator analyses investigating the influence of number of loads on model accuracy using both scaled and unscaled residuals.

| **Residuals (kg)** | | | | | **Residuals scaled (%1RM)** | | | |
| --- | --- | --- | --- | --- | --- | --- | --- | --- |
| *Predictors* | *Estimates* | | *95% CI* | *p* | | *Estimates* | *95%CI* | *p* |
| Two (intercept) | 5.9 | | 2.7 to 9.0 | 0.003 | | 5.1 | -2.0 to 8.1 | 0.004 |
| Multiple | -1.5 | | -3.0 to –0.1 | 0.038 | | 1.3 | -3.7 to 1.1 | 0.236 |
|  |  | |  |  | |  |  |  |
| **Random effects** | | | | | | | | |
| σ | | 10.7 | | | 7.9 | | | |
| τ_within_ | | 6.3 | | | 4.4 | | | |
| τ_between_ | | 4.6 | | | 5.3 | | | |
| Observations | | 2234 (100 models; 16 studies) | | | 2168 (94 models; 15 studies) | | | |

**Table 4:** Results of moderator analyses investigating influence of group or individualised MVT’s on model accuracy using both scaled and unscaled residuals.

| **Residuals (kg)** | | | | | **Residuals scaled (%1RM)** | | | |
| --- | --- | --- | --- | --- | --- | --- | --- | --- |
| *Predictors* | *Estimates* | | *95% CI* | *p* | | *Estimates* | *95%CI* | *p* |
| Group (intercept) | 4.3 | | 0.9 to 7.6 | 0.018 | | 4.5 | 0.4 to 8.5 | 0.0339 |
| Individual | 1.4 | | -2.0 to 4.9 | 0.301 | | 0.1 | -2.4 to 2.7 | 0.904 |
| Vlast | 0.8 | | -1.2 to 2.8 | 0.291 | | 0.6 | -0.8 to 2.0 | 0.262 |
|  |  | |  |  | |  |  |  |
| **Random effects** | | | | | | | | |
| σ | | 8.6 | | | 7.3 | | | |
| τ_within_ | | 5.0 | | | 4.3 | | | |
| τ_between_ | | 5.6 | | | 5.8 | | | |
| Observations | | 1876 (76 models; 14 studies) | | | 1810 (70 models; 13 studies) | | | |

References

BALSALOBRE-FERNANDEZ, C. et al., 2018. Validity and reliability of a novel iPhone app for the measurement of barbell velocity and 1RM on the bench-press exercise. *Journal of Sports Sciences*, 36(1), pp. 64–70.

BERTON, R. et al., 2021. Concurrent validity and reliability of the load-velocity relationship to predict the one-repetition maximum during three weightlifting derivatives. *Kinesiology*, 53(2), pp. 215–225.

BISHOP, D. et al., 2020. A preliminary investigation into the validity of a submaximal protocol to predict one repetition maximum (1-RM) in the back squat, (Journal Article PG-). [online]. Available from: NS -.

CALLAGHAN, D. et al., 2019. VALIDATION OF TWO MOBILE APPS TO PREDICT MAXIMAL STRENGTH. *ISBS Proceedings Archive*, 37(1 PG-511), p. 511.

CAVEN, E.J.G. et al., 2020. Group versus Individualised Minimum Velocity Thresholds in the Prediction of Maximal Strength in Trained Female Athletes. *International Journal of Environmental Research and Public Health*, 17(21 PG-). [online]. Available from: https://ezproxy.rgu.ac.uk/login?url=https://search.ebscohost.com/login.aspx?direct=true&AuthType=ip,shib&db=cmedm&AN=33114479&site=ehost-live&scope=sitehttps://ezproxy.rgu.ac.uk/login?url=https://search.ebscohost.com/login.aspx?direct=true&AuthType=ip,shi.

JANICIJEVIC, D. et al., 2020. Bench Press 1-Repetition Maximum Estimation Through the Individualized Load–Velocity Relationship: Comparison of Different Regression Models and Minimal Velocity Thresholds. *International Journal of Sports Physiology and Performance*, pp. 1–8.

JIMÉNEZ-ALONSO, A. et al., 2020. Velocity Performance Feedback During the Free-Weight Bench Press Testing Procedure: An Effective Strategy to Increase the Reliability and One Repetition Maximum Accuracy Prediction. *Journal of Strength and Conditioning Research*, (Journal Article PG-). [online]. Available from: https://ezproxy.rgu.ac.uk/login?url=https://search.ebscohost.com/login.aspx?direct=true&AuthType=ip,shib&db=cmedm&AN=32282530&site=ehost-live&scope=sitehttps://ezproxy.rgu.ac.uk/login?url=https://search.ebscohost.com/login.aspx?direct=true&AuthType=ip,shi.

JUKIC, I. et al., 2022. Validity of load–velocity relationship to predict 1 repetition maximum during deadlifts performed with and without lifting straps: The accuracy of six prediction models. *Journal of Strength and Conditioning Research*, 36(4), pp. 902–910.

KILGALLON, J. et al., 2022. Reliability and validity of velocity measures and regression methods to predict maximal strength ability in the back-squat using a novel linear position transducer. *Proceedings of the Institution of Mechanical Engineers Part P Journal of Sports Engineering and Technology*.

MACARILLA, C.T., 2020. The Accuracy of Predicting One-repetition Maximum from Submaximal Velocity in the Back Squat and Bench Press, (Generic PG-). [online]. Available from: NS -.

NICKERSON, B.S. et al., 2020. Evaluation of Load-Velocity Relationships and Repetitions-to-Failure Equations in the Presence of Male and Female Spotters. *Journal of Strength and Conditioning Research*, 34(9), pp. 2427–2433.

PEREZ-CASTILLA, A. et al., 2019. Precision of 7 Commercially Available Devices for Predicting Bench-Press 1-Repetition Maximum From the Individual Load-Velocity Relationship. *International Journal of Sports Physiology and Performance*, 14(10 PG-1442–1446), pp. 1442–1446.

RUF, L., CHERY, C. and TAYLOR, K.-L., 2018. Validity and Reliability of the Load-Velocity Relationship to Predict the One-Repetition Maximum in Deadlift. *Journal of Strength and Conditioning Research*, 32(3 PG-681–689), pp. 681–689.

THOMPSON, S.W. et al., 2021. A Novel Approach to 1RM Prediction Using the Load-Velocity Profile: A Comparison of Models. *Sports*, 9(7).

WILLIAMS, T.D. et al., 2020. Bench Press Load-Velocity Profiles and Strength After Overload and Taper Microcyles in Male Powerlifters. *Journal of Strength and Conditioning Research*, (Journal Article PG-). [online]. Available from: https://ezproxy.rgu.ac.uk/login?url=https://search.ebscohost.com/login.aspx?direct=true&AuthType=ip,shib&db=cmedm&AN=33021581&site=ehost-live&scope=sitehttps://ezproxy.rgu.ac.uk/login?url=https://search.ebscohost.com/login.aspx?direct=true&AuthType=ip,shi.
